# Characterization of Methicillin-Resistant *Staphylococcus aureus* Isolated from Healthy Turkeys and Broilers Using DNA Microarrays

**DOI:** 10.3389/fmicb.2016.02019

**Published:** 2016-12-19

**Authors:** Hosny El-Adawy, Marwa Ahmed, Helmut Hotzel, Stefan Monecke, Jochen Schulz, Joerg Hartung, Ralf Ehricht, Heinrich Neubauer, Hafez M. Hafez

**Affiliations:** ^1^Friedrich-Loeffler-Institut, Institute of Bacterial Infections and ZoonosesJena, Germany; ^2^Department of Poultry Diseases, Faculty of Veterinary Medicine, Kafrelsheikh UniversityKafr El-Sheikh, Egypt; ^3^Institute for Animal Hygiene, Animal Welfare and Farm Animal Behaviour, University of Veterinary Medicine Hannover, FoundationHannover, Germany; ^4^Department of Animal Hygiene and Zoonoses, Faculty of Veterinary Medicine, Mansoura UniversityMansoura, Egypt; ^5^Alere Technologies GmbHJena, Germany; ^6^InfectoGnostics Research Campus Jena e. V.Jena, Germany; ^7^Medical Faculty Carl Gustav Carus, Institute for Medical Microbiology and Hygiene, Technische Universität DresdenDresden, Germany; ^8^Institute for Poultry Diseases, Free University BerlinBerlin, Germany

**Keywords:** *Staphylococcus aureus*, MRSA, turkey, broiler, microarray, genotyping, antibiotics

## Abstract

Methicillin-resistant *Staphylococcus aureus* (MRSA) is a major human health problem and recently, domestic animals are described as carriers and possible reservoirs. Twenty seven *S. aureus* isolates from five turkey farms (*n* = 18) and two broiler farms (*n* = 9) were obtained by culturing of choana and skin swabs from apparently healthy birds, identified by Taqman-based real-time duplex *nuc*-*mec*A-PCR and characterized by *spa* typing as well as by a DNA microarray based assay which covered, amongst others, a considerable number of antibiotic resistance genes, species controls, and virulence markers. The antimicrobial susceptibility profiles were tested by agar diffusion assays and genotypically confirmed by the microarray. Five different *spa* types (3 in turkeys and 2 in broilers) were detected. The majority of MRSA isolates (24/27) belonged to clonal complex 398-MRSA-V. The most frequently occurring *spa* types were accordingly t011, t034, and t899. A single CC5-MRSA-III isolated from turkey and CC398-MRSA with an unidentified/truncated SCC*mec* element in turkey and broiler were additionally detected. The phenotypic antimicrobial resistance profiles of *S. aureus* isolated from both turkeys and broilers against 14 different antimicrobials showed that all isolates were resistant to ampicillin, cefoxitin, oxacillin, doxycycline, and tetracycline. Moreover, all *S. aureus* isolated from broilers were resistant to erythromycin and azithromycin. All isolates were susceptible to gentamicin, chloramphenicol, sulphonamides, and fusidic acid. The resistance rate against ciprofloxacin was 55.6% in broiler isolates and 42.1% in turkey isolates. All tetracycline resistant isolates possessed genes *tet*K/M. All erythromycin-resistant broiler isolates carried *ermA*. Only one broiler isolate (11.1%) carried genes *ermA, ermB*, and *ermC*, while 55.6% of turkey isolates possessed *erm*A and *erm*B genes. Neither PVL genes (*luk*F/S-PV), animal-associated leukocidin (*luk*M and *luk*-P83) nor the gene encoding the toxic shock syndrome toxin (*tst*1) were found in turkey and broiler isolates. In conclusion, the detection of MRSA in healthy turkeys and broilers with even additional antibiotic resistance markers is of major public health concern. The difference in antibiotic resistance and virulence markers between MRSA isolates from turkeys and broilers was addressed.

## Introduction

*Staphylococcus aureus* is considered as one of the most prevalent agents causing food intoxication worldwide resulting from ingestion of heat-stable staphylococcal enterotoxins (Le Loir et al., 2003). Recently, methicillin-resistant *Staphylococcus aureus* (MRSA) has been increasingly reported as an emerging problem in veterinary medicine (Leonard and Markey, 2008).

Antimicrobial agents, including penicillin, erythromycin, and tetracyclines, are widely used for treating staphylococcal and other infections in poultry (White et al., 2003).

Although detection and genotyping of *S. aureus* was common in diseased poultry (Richter et al., 2012; Argudín et al., 2013; Monecke et al., 2013; Kraushaar et al., 2016) and from poultry meat (Boost et al., 2013; Buyukcangaz et al., 2013; Krupa et al., 2014; Abdalrahman et al., 2015; Kim et al., 2015; Sallam et al., 2015; Bortolaia et al., 2016; Kraushaar et al., 2016; Raji et al., 2016), characterization data of *S. aureus* isolates from healthy turkeys and broilers are still widely lacking.

Investigations of the genetic relatedness and differentiation of MRSA isolates in local and global epidemiological studies were carried out using results of MLST, *spa* typing and characterization of staphylococcal cassette chromosome *mec* (SCC*mec*) elements (Grundmann et al., 2010).

DNA microarrays based assays facilitating rapid detection of resistance determinants, toxin genes, a variety of virulence-associated determinants and other typing markers have recently been applied for *S. aureus* (Monecke et al., 2007). Microarrays allow high-throughput, accurate, rapid and economic assignment of MRSA to sequence types (STs), clonal complexes (CCs), and SCC*mec* types and provide further evidence of the diversity of SCC*mec*/SCC elements (Shore et al., 2011).

MRSA originating from animals especially pigs or turkeys usually belonged to clonal complex CC398 (Richter et al., 2012). MRSA of CC398 and/or CC9 have been identified in healthy broilers (Nemati et al., 2008) as well as in diseased broilers and turkeys (Monecke et al., 2013) and were also found in food and food products of broiler and turkey origin with wide variability of resistance phenotypes and genotypes (Feßler et al., 2011). Moreover, CC5-MRSA-III has been identified in diseased chickens and turkeys in Germany (Monecke et al., 2013).

Most MRSA isolates are resistant to several classes of antimicrobial substances. More than 80% of these strains produce penicillinases responsible for penicillin resistance. Resistance to all other beta-lactam antibiotics is usually mediated by the *mecA* or *mecC* gene (Leonard and Markey, 2008; Shore et al., 2011).

*S. aureus* isolated from diseased turkeys showed predominantly resistance to erythromycin, tetracycline and methicillin (Jaglic et al., 2012).

Risk factors for MRSA infection in poultry are currently under investigation and such data are essential for the preparation of specific guidelines for control of MRSA in veterinary practice.

The objective of this study was the analysis of MRSA isolates from apparently healthy turkey and broiler farms with particular reference to some genotypic characteristics, including virulence determinants and their antimicrobial resistance patterns using microarray analysis.

## Materials and methods

### Bacterial isolates

In 2013, five turkey farms (25,450 birds) and two broiler farms (13,200 birds) in southwest and central Germany were screened for MRSA during the production cycle. The samples were collected from apparently healthy birds in the farms. Briefly, 60 skin swabs samples and 60 choana swabs were collected from each farm. In case of broiler, samples were taken from skin under one wing, and turkey samples were obtained from skin of the neck, using cotton swabs (Sarstedt AG & Co KG, Nümbrecht, Germany) wetted with sterile phosphate-buffered saline. For collecting samples from the choana, dry cotton swabs (nerbe plus GmbH, Winsen, Germany) were used. Swabs were streaked directly onto selective agar (CHROMagar MRSA, MAST Diagnostica GmbH, Reinfeld, Germany) and incubated under aerobic conditions for 24 h at 37°C. Additionally, the swabs were incubated in 10 ml of Mueller-Hinton broth (CM0405; Oxoid Ltd., Hampshire) with 6% NaCl for 24 h at 37°C. Following incubation, 1 ml of the enrichment suspension was added to 9 ml tryptone soy broth (CM0129; Oxoid Ltd., Hampshire) containing 75 mg/l aztreonam and 3.5 mg/l cefoxitin (TSB+) to grow MRSA aerobically for 17 h at 37°C. A loopful of TSB+ was streaked onto selective agar and incubated for 24 h at 37°C. Characteristic MRSA colonies were identified by coagulase test (Becton Dickinson, Heidelberg, Germany). Taqman-based real-time duplex *nuc*-*mec*A-PCR were used to detect MRSA (Pasanen et al., 2010).

The *spa* typing was performed according to previously published protocols using primers spa-1113f and spa-1514r (Hasman et al., 2010) and alternative primers spa-1095f and spa-1517r (Votintseva et al., 2014; Holtfreter et al., 2016). Chromatograms were analyzed using Ridom StaphType v2.0.3 software (Ridom GmbH). The relationships between *spa*-types were investigated using the BURP clustering algorithm (Mellmann et al., 2007) incorporated into Ridom StaphType (www.SeqNet.org).

### Ethics statement

This study was carried out in strict accordance with the recommendations in the Guide for the Care and Use of Laboratory Animals of the University of Veterinary Medicine Hannover. The protocol was approved by the Animal Welfare Officer of the University. All efforts were made to minimize animal suffering and to reduce the number of animals used.

### Antimicrobial susceptibility testing

Isolates were subjected to phenotypic antimicrobial susceptibility profiling against 14 antimicrobials that belonged to eight different antibiotic classes (Tables 1, 2) using agar diffusion tests. Isolates were grown on Mueller-Hinton (MH) agar (Oxoid Ltd., Hampshire) and incubated for 16–20 h at 37°C. Cultures were added to Mueller-Hinton broth (Oxoid Ltd., Hampshire), adjusted to a turbidity equal to a 0.5 McFarland standard, and inoculated onto 6-inch Mueller-Hinton agar plates supplemented with the appropriate antimicrobial at different concentrations (Tables 1, 2) including the breakpoint established for each antimicrobial according to the Clinical and Laboratory Standards Institute (CLSI, 2015). Plates were incubated up to 16–20 h at 37°C and results were read for growth or no growth and denoted as resistant or susceptible (Tables 1, 2). The reference strain *S. aureus* ATCC 29213 was used as quality control in the MIC determinations.

**Table 1:**
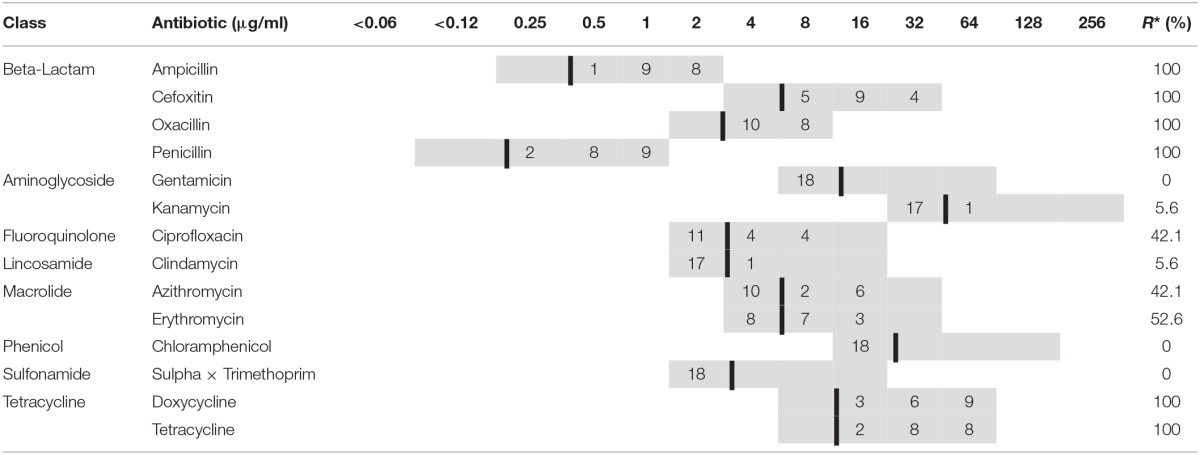
**Antibiotic susceptibility testing of 18 ***S. aureus*** isolated from turkeys to 14 antimicrobial agents using agar gel diffusion test**.

*A thick black line indicates the break point between clinically sensitive and resistant strains. A gray shadowed area indicates the test range (μg/ml) of each antimicrobial agent*.

*R, resistance rate*.

**Table 2:**
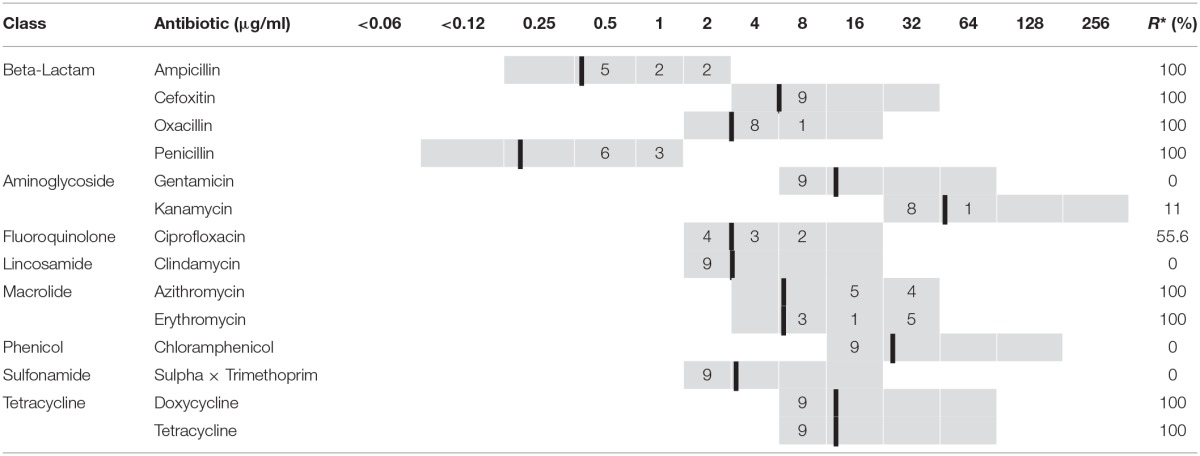
**Antibiotic susceptibility testing of 9 ***S. aureus*** isolated from broilers to 14 antimicrobial agents using agar gel diffusion test**.

*A thick black line indicates the break point between clinically sensitive and resistant strains. A gray shadowed area indicates the test range (μg/ml) of each antimicrobial agent*.

*R, resistance rate*.

### DNA microarray analysis

Gene loci responsible for the antibiotic resistance in addition to genes that are associated with virulence of the *S. aureus* isolates as well as genes encoding toxins and adhesion factors were detected using the *S. aureus* specific diagnostic DNA microarray based assay (StaphyType, Alere Technologies GmbH, Jena, Germany). The StaphyType DNA microarrays cover 333 target sequences which correspond to approximately 170 distinct genes and their allelic variants. These include species markers, SCC*mec* elements, capsule, and accessory gene regulator (*agr*) group typing markers, resistance genes, and genes encoding exotoxins as well as adhesion factors (Data sheet 1). Arrays were evaluated, verified and validated side by side with PCR and next generation sequencing (NGS) extensively (Monecke et al., 2007, 2011; Shore et al., 2012).

The detection method and primer/probe sequences have been described previously in detail (Monecke et al., 2011). Briefly, *S. aureus* were sub-cultivated on Columbia blood agar (Oxoid Ltd., Hampshire), harvested and lysed. DNA was prepared utilizing spin columns or the automated EZ1 system (Qiagen, Hilden, Germany). DNA samples were subjected to a linear primer elongation using only one primer per target. During this step, biotin-16-dUTP was incorporated into the resulting amplicons. In a later step, these single stranded DNA amplicons were hybridized to the probes of the microarray. After washing, horseradish-peroxidase-streptavidin conjugate was added which subsequently triggered the precipitation of a dye. An image of the microarray was taken and automatically analyzed using a reader and dedicated software provided by Alere Technologies GmbH. The automated comparison of the hybridization patterns of the actual isolate to a reference database allowed determining its affiliation to clonal complexes as defined by MLST (Enright et al., 2000) and, in case of MRSA, to epidemic strains defined by MLST and SCC*mec* carriage (Monecke et al., 2011).

## Results

### Molecular characterization of bacterial isolates

Twenty seven methicillin-resistant *S. aureus* (18 from turkeys and nine from broilers) were isolated and identified by microbiological, molecular biological methods and species markers covered by the microarray assay (Table 3). They showed positive hybridization results with gene probes for domain 1 of 23S-rRNA, catalase A (*kat*A), coagulase (*co*A) and glyceraldehyde 3-phosphate dehydrogenase locus. All isolates possessed the *mec*A gene characterizing MRSA isolates (Table 3). The gene *mecC* was absent from all isolates.

**Table 3:** **Genotyping of MRSA isolates from turkeys and broilers**.

**Species**	**Sample**	**Clonal complex**	***spa-*****Type**	**Methicillin resistance and** ***SCC*****mec typing**													
				***mec*****A**	**delta_*mec*R**	***ugp*****Q**	***ccr*****A-1**	***ccr*****B-1**	**Q9XB68-dcs**	***xyl*****R**	***ccr*****A-3**	***ccr*****B-3**	***ccr*****AA probe 1**	***ccr*****AA probe 2**	***ccr*****C**	***ccr*****A-4**	***ccr*****B-4**
Turkey	Choana swab	CC389 MRSA-V	t011	POS	NEG	POS	NEG	NEG	NEG	NEG	NEG	NEG	POS	POS	POS	NEG	NEG
Turkey	Choana swab	CC389 MRSA-V	t011	POS	NEG	POS	NEG	NEG	NEG	NEG	NEG	NEG	POS	POS	POS	NEG	NEG
Turkey	Choana swab	CC389 MRSA-V	t011	POS	NEG	POS	NEG	NEG	NEG	NEG	NEG	NEG	POS	POS	POS	NEG	NEG
Turkey	Choana swab	CC389 MRSA-V	t011	POS	NEG	POS	NEG	NEG	NEG	NEG	NEG	NEG	POS	POS	POS	NEG	NEG
Turkey	Choana swab	CC389 MRSA-V	t011	POS	NEG	POS	NEG	NEG	NEG	NEG	NEG	NEG	POS	POS	POS	NEG	NEG
Turkey	Choana swab	CC389 MRSA-V	t034	POS	NEG	POS	NEG	AMB	NEG	NEG	NEG	NEG	POS	POS	POS	NEG	NEG
Turkey	Choana swab	CC389 MRSA-V	t034	POS	NEG	POS	NEG	NEG	NEG	NEG	NEG	NEG	POS	POS	POS	NEG	NEG
Turkey	Choana swab	CC389 MRSA-V	t034	POS	NEG	POS	NEG	AMB	NEG	NEG	NEG	NEG	POS	POS	POS	NEG	NEG
Turkey	Choana swab	CC389 MRSA-V	t034	POS	NEG	POS	NEG	AMB	NEG	NEG	NEG	NEG	POS	POS	POS	NEG	NEG
Turkey	Skin swab	CC389 MRSA-V	t011	POS	NEG	POS	NEG	NEG	NEG	NEG	NEG	NEG	POS	POS	POS	NEG	NEG
Turkey	Skin swab	CC389 MRSA-V	t011	POS	NEG	POS	NEG	NEG	NEG	NEG	NEG	NEG	POS	POS	POS	NEG	NEG
Turkey	Skin swab	CC389 MRSA-V	t034	POS	NEG	POS	NEG	NEG	NEG	NEG	NEG	NEG	POS	POS	POS	NEG	NEG
Turkey	Skin swab	CC389 MRSA-V	t011	POS	NEG	POS	NEG	AMB	NEG	NEG	NEG	NEG	POS	POS	POS	NEG	NEG
Turkey	Skin swab	CC389 MRSA-V	t011	POS	NEG	POS	NEG	NEG	NEG	NEG	NEG	NEG	POS	POS	POS	NEG	NEG
Turkey	Skin swab	CC389 MRSA-V	t034	POS	NEG	POS	NEG	AMB	NEG	NEG	NEG	NEG	POS	POS	POS	NEG	NEG
Turkey	Skin swab	CC389 MRSA-V	t034	POS	NEG	POS	NEG	NEG	NEG	NEG	NEG	NEG	POS	POS	POS	NEG	NEG
Turkey	Skin swab	CC389 MRSA-n.t.^#^	n.t.^#^	POS	POS	POS	NEG	NEG	AMB	NEG	NEG	NEG	POS	POS	POS	NEG	NEG
Turkey	Skin swab	CC5 MRSA-III	t002	POS	POS	POS	NEG	AMB	POS	NEG	POS	POS	NEG	NEG	NEG	NEG	NEG
Broiler	Choana swab	CC389 MRSA-n.t.^#^	n.t.^#^	POS	POS	POS	NEG	NEG	POS	NEG	NEG	NEG	POS	POS	POS	NEG	NEG
Broiler	Choana swab	CC389 MRSA-V	t899	POS	NEG	POS	NEG	NEG	NEG	NEG	NEG	NEG	POS	POS	POS	NEG	NEG
Broiler	Choana swab	CC389 MRSA-V	t899	POS	NEG	POS	NEG	AMB	NEG	NEG	NEG	NEG	POS	POS	POS	NEG	NEG
Broiler	Choana swab	CC389 MRSA-V	t899	POS	NEG	POS	NEG	AMB	NEG	NEG	NEG	NEG	POS	POS	POS	NEG	NEG
Broiler	Choana swab	CC389 MRSA-V	t899	POS	NEG	POS	NEG	AMB	NEG	NEG	NEG	NEG	POS	POS	POS	NEG	NEG
Broiler	Choana swab	CC389 MRSA-V	t899	POS	NEG	POS	NEG	NEG	NEG	NEG	NEG	NEG	POS	POS	POS	NEG	NEG
Broiler	Skin swab	CC389 MRSA-V	t899	POS	NEG	POS	NEG	AMB	NEG	NEG	NEG	NEG	POS	POS	POS	NEG	NEG
Broiler	Skin swab	CC389 MRSA-V	t899	POS	NEG	POS	NEG	AMB	NEG	NEG	NEG	NEG	POS	POS	POS	NEG	NEG
Broiler	Skin swab	CC389 MRSA-V	t1430	POS	NEG	POS	NEG	AMB	NEG	NEG	NEG	NEG	POS	POS	POS	NEG	NEG

*POS, Positive; NEG, Negative; AMB, Ambiguous. mecA, alternate penicillin binding protein 2, defining MRSA; delta_mecR, truncated signal transducer protein MecR1; ugpQ, glycerol-phosphoryl-diester-phospho-di-esterase, associated with mecA; ccrA-1; ccrB-1, cassette chromosome recombinase genes A/B-1; Q9XB68-dcs, hypothetical protein from SCCmec elements; Q9XB68-dcs; xylR, homolog of xylose repressor, associated with SCCmec-elements; ccrA-3; ccrB-3, cassette chromosome recombinase genes A/B-3; ccrAA (MRSAZH47)_probe 1; ccrAA (MRSAZH47)_probe 2; ccrC (85–2082), cassette chromosome recombinase genes “ccrAA” (hypothetical) and ccrC; ccrA-4; ccrB-4; cassette chromosome recombinase genes A/B-4*.

# *not typed*.

In total, five different *spa* types of MRSA were found (3 from turkey farms and two from broilers). Additionally, two isolates (from broiler and turkey) were not *spa* typable because no successful amplification was achieved (Table 3). The majority of isolates from turkeys are belonged to *spa* type t011 (9 out of 18 = 50.0%), followed by t034 (6 out of 18 = 33.3%). In addition, one isolate from turkey belonged to *spa* t002. Concerning the nine isolates from broilers, seven belonged to *spa* t899 (77.7%), one to *spa* t1430 and one was non-assignable as in this isolate *spa* PCR sequence product was not in database either by spa-1113f and spa-1514r or spa-1095f and 1517r primers. The majority of investigated MRSA isolates (24/27; 88.9%) belonged to the CC398-MRSA-V strain carrying *spa* types t899, t1430, t011 and t034. Two isolates (turkey and broiler) belonged to CC398-MRSA with a yet unidentified/truncated SCC*mec* element. Furthermore, a single CC5-MRSA-III of *spa* type t002 was isolated from skin swab of a turkey (Table 3).

### Antimicrobial susceptibility profiles

All isolates from turkeys and broilers were resistant to ampicillin, cefoxitin, oxacillin, penicillin, doxycycline, and tetracycline. In addition, all *S. aureus* isolated from broilers were resistant to azithromycin and erythromycin. All isolates were susceptible to gentamicin, chloramphenicol, and sulphonamides (Tables 1, 2). The resistance rate of 18 *S. aureus* isolates from turkeys to erythromycin, azithromycin, ciprofloxacin, clindamycin and kanamycin were 52.6, 42.1, 42.1, 5.6, 5.6%, respectively (Table 1). While, the resistance rate of 9 *S. aureus* isolates from broilers to ciprofloxacin and kanamycin were 55.6 and 11.0%, respectively (Table 2). The resistance rates for azithromycin, ciprofloxacin, kanamycin, clindamycin, and erythromycin were higher among the broiler isolates than for turkey isolates.

### Susceptibility tests and detection of antibiotic resistance genes

Prevalence of antibiotic resistance genes varied among the isolates from turkeys and broilers (Table 3). All 27 MRSA from turkeys and broilers carried tetracycline resistance genes *tetK* and *tetM*. All MRSA isolated from broilers carried the erythromycin resistance gene *ermA*, while *ermB* and *ermC* were detected once only. 55.6% of turkey isolates were resistant to erythromycin and carried resistance genes *ermA* and *ermB*. A detailed overview about detected genes responsible for antimicrobial resistance is shown in Table 4.

**Table 4:** **Antibiotic resistance of ***S. aureus*** isolates based on detection of specific genes**.

**Antibiotic resistance genes**		**Turkey**		**Broiler**	
		**Choana swab (*n* = 9)**	**Skin swab (*n* = 9)**	**Choana swab (*n* = 6)**	**Skin swab (*n* = 3)**
Gentamicin	*aacA-aphD*	0 (0%)	0 (0%)	0 (0%)	0 (0%)
Tobramycin	*AadD*	6 (66. 66%)	3 (33. 33%)	1 (16. 66%)	0 (0%)
Neo-/Kanamycin	*aphA3*	0 (0%)	1 (11. 11%)	0 (0%)	0 (0%)
Streptothricine	*Sat*	0 (0%)	1 (11. 11%)	0 (0%)	0 (0%)
Sulpha/Trimethoprim	*dfr2S1*	0 (0%)	0 (0%)	0 (0%)	0 (0%)
Fusidic acid	*far1*	0 (0%)	0 (0%)	0 (0%)	0 (0%)
Tetracycline	*tet(K)*	9 (100%)	9 (100%)	6 (100%)	3 (100%)
	*tet(M)*	9 (100%)	9 (100%)	6 (100%)	3 (100%)
Chloramphenicol	*Cat*	0 (0%)	0 (0%)	0 (0%)	0 (0%)
Fosfomycin	*FosB*	6 (66. 66%)	4 (44. 44%)	3 (50%)	2 (66. 66%)
	*fosB* (plasmid)	1 (11. 11%)	0 (0%)	0 (0%)	0 (0%)
Penicillin	*BlaZ*	9 (100%)	9 (100%)	6 (100%)	3 (100%)
	*BlaI*	9 (100%)	9 (100%)	6 (100%)	3 (100%)
	*BlaR*	9 (100%)	9 (100%)	6 (100%)	3 (100%)
Erythromycin/ clindamycin	*ErmA*	3 (33. 33%)	4 (44. 44%)	6 (100%)	3 (100%)
	*ErmB*	6 (66. 66%)	3 (33. 33%)	1 (11. 11%)	0 (0%)
	*ErmC*	0 (0%)	0 (0%)	1 (11. 11%)	0 (0%)
	*MsrA*	0 (0%)	0 (0%)	0 (0%)	0 (0%)
	*MefA*	0 (0%)	0 (0%)	0 (0%)	0 (0%)
Lincosamide	*LnuA*	0 (0%)	1 (11. 11%)	0 (0%)	0 (0%)
Macrolides	*MphC*	0 (0%)	1 (11. 11%)	0 (0%)	0 (0%)
Streptogramin	*VatA*	0 (0%)	0 (0%)	0 (0%)	0 (0%)
	*VatB*	0 (0%)	0 (0%)	0 (0%)	0 (0%)
	*VgaA*	0 (0%)	0 (0%)	0 (0%)	0 (0%)
	*vgaA* (BM 3327)	6 (66. 66%)	5 (55. 55%)	0 (0%)	0 (0%)
	*VgbA*	0 (0%)	0 (0%)	0 (0%)	0 (0%)

*aacA-aphD, aminoglycoside acetyltransferase A/aminoglycoside phosphotransferase D gene; aadD, aminoglycoside adenyltransferase D gene; aphA3, aminoglycoside phosphotransferase type III gene; sat, strepto-thricine-acetyl-transferase gene; dfrS1, dihydro-folate reductase type 1 gene; far1, fusidic acid resistance encoding gene; tet, tetracycline-resistance encoding gene; cat, chloramphenicol acetyltransferase gene; fosB, fosB (plasmid): metallothiol transferase gene; blaZ, beta-lactamase; blaI, beta lactamase repressor (inhibitor) gene; blaR, beta-lactamase regulatory protein gene; ermA, ermB, ermC, rRNA adenine N-6-methyl-transferase gene; lnuA, lincosamid-nucleotidyltransferase gene; msrA, energy-dependent efflux protein gene; mefA, macrolide efflux protein A gene; mphC, lysylphosphatidylglycerol synthetase/glycosyltransferase gene; vatA, virginiamycin A acetyltransferase gene; vatB, acetyltransferase gene; vgaA, vgaA (BM 3327), ATP binding protein gene; vgbA, virginiamycin B lyase gene*.

Only one MRSA isolate from a skin swab of a turkey carried the resistant gene *lnuA* which mediates resistance against lincosamide and it carried also the macrolide inactivation gene *mphC*.

Eleven out of 18 MRSA isolates (61.1%) from turkeys were resistant to streptogramin and carried the resistance gene *vgaA* encoding for an ATP binding protein. In contrast, no broiler isolate was a carrier of streptogramin resistance determinants.

In one turkey isolate, *sat* and *aphA3* genes were detected which are responsible for resistance against streptothricin and neomycin/kanamycin, respectively. With regard to tobramycin resistance, 50.0% of turkey isolates and 11.1% of broiler isolates were found to harbor *aadD*.

Multidrug resistance as defined as resistance against at least three different classes of antibiotics was detected in MRSA isolates from both, turkeys and broilers (Table 5). All tested isolates were showed to be resistance to tetracycline and penicillin. All broiler isolates were additionally resistant to erythromycin, while seven of turkey isolates (38.9%) possessed *ermA* and 9 (50.0%) *ermB* genes. One turkey isolate assigned to CC5-MRSA-III was resistant to penicillin, neomycin, kanamycin, streptothricine, erythromycin, fosfomycin, and tetracycline.

**Table 5:** **Relationship between phenotypic antimicrobial resistance and detection of resistance genes in MRSA isolated from turkeys and broilers**.

**Antimicrobial agent**	**Phenotypic resistance**		**Genotypic resistance**		
	**Turkeys (%)**	**Broilers (%)**	**Resistance genes**	**Turkeys (%)**	**Broilers (%)**
Beta-lactam	100	100	*mecA, blaZ/I/R*	100	100
Tetracycline	100	100	*tetK, tetM*	100	100
Erythromycin	52.6	100	*ermA, ermB, ermC*	55.6	100
Fosfomycin	ND	ND	*fosB*	61.1	55.5
Streptogramin	ND	ND	*vgaA*	61.1	0
Tobramycin	ND	ND	*aadD*	50	11.1
Streptothricin	ND	ND	*sat*	11.1	0
Lincosamide	5.6	0	*lnuA*	5.6	0
Neomycin	ND	ND	*aphA3*	5.5	0
Kanamycin	5.2	11	*aphA3*	5.5	0
			*aadD*	0	11.1
Gentamicin	0	0	*aacA-aphD*	0	0
Chloramphenicol	0	0	*cat*	0	0
Sulphonamides	0	0	*dfrS1*	0	0
Fucidic acid	ND	ND	*far1*	0	0

*ND, not done*.

The concordance between the measured genes and the antimicrobial phenotypes is shown in Table 5.

### Presence of virulence-associated genes

A summary of the carriage of virulence-associated factors is provided in Table 6. The genes encoding the toxic shock syndrome toxin (*tst*1), *sea, seb, sec*, and *see*, the epidermal cell differentiation inhibitor genes *edin*A/B/C and genes for exfoliative toxins *etA*/*B* were not found in any turkey and broiler isolates. Panton—Valentine leukocidin (*PVL*) or *lukM*/*lukF*-*PV*(P83) genes were also not detected.

**Table 6:** **Virulence associated genes of ***S. aureus*** isolates**.

**Staphylococcal super-antigen/enterotoxin-like genes (*set*/*ssl*)**		**Turkey**		**Broiler**	
		**Choana swab (*n* = 9)**	**Skin swab (*n* = 9)**	**Choana swab (*n* = 6)**	**Skin swab (*n* = 3)**
Staphylococcal super-antigen-like protein 1	ssl01/set6 p.2/11	2	1	0	0
	ssl01/set6 p.1/12	9	9	6	3
	ssl01/set6 p.4/11	9	9	4	3
	ssl01/set6 (COL)	6	5	3	2
	ssl01/set6 (Mu50+N315)	3	4	3	1
	ssl01/set6 (MRSA252)	2	1	0	0
Staphylococcal super-antigen-like protein 2	ssl02/set7	0	1	0	0
	ssl02/set7 (MRSA252)	9	8	6	3
Staphylococcal super-antigen-like protein 3	ssl03/set8 P.1/2	0	1	0	0
	ssl04/set9	2	2	0	0
	ssl04/set9	9	8	4	2
Staphylococcal super-antigen-like protein 4	ssl05/set3 p. 1	0	1	0	0
	ssl05/set3 p.1	8	5	3	3
Staphylococcal super-antigen-like protein 5	ssl05/set3p.2	0	1	0	0
	ssl05/set3 MRSA252	9	8	6	3
	ssl06 NCTC8325+MW2	7	3	2	2
Staphylococcal super-antigen-like protein 6	ssl07/set1	0	2	0	0
	ssl07/set1 MRSA252	9	7	4	3
Staphylococcal super-antigen-like protein 7	ssl07/set1 AF188836	0	0	2	0
	ssl08/set12 p.1	9	8	6	3
Staphylococcal super-antigen-like protein 8	ssl08/set12 p. 1/2	0	1	0	0
Staphylococcal super-antigen-like protein 9	ssl09/set5 P. 1/2	0	1	0	0
	ssl09/set5 MRSA252	9	8	6	3
Staphylococcal super-antigen-like protein 10	ssl10/ set4	9	8	3	1
	ssl10/set4 MRSA252	0	1	3	2
Staphylococcal super-antigen-like protein 11	ssl11+set2Mu50+N315	0	1	0	0
	ssl11/set2 MRSA252	9	8	4	3
Staphylococcal exotoxin-like protein, second locus	setB3	0	1	0	0
	setB2	0	1	0	0
	setB2 MRSA252	9	8	6	3

The *egc* enterotoxin gene cluster which comprises the enterotoxin G, I, M, N, O, and U genes (*se*g, *se*i, *selm, seln, selo, selu*) was found once in strain isolated from turkey skin which belonged to CC5 MRSA-III. Hemolysin and leukocidin genes (*hlgA, lukF, lukS, lukX, lukY*) were detected in all isolates. The leucocidin genes *lukD* and *lukE* and hemolysin factor *hlb* were detected only in to the CC5-MRSA-III isolate.

Staphylococcal superantigen/enterotoxin-like genes (SET/SSL) were variably detected in turkey and broiler isolates yielding hybridization patterns corresponding to the respective clonal complexes (Table 6).

The adhesion factors and genes encoding microbial surface components recognizing adhesive matrix molecules [bone binding protein (*bbp*), clumping factors (*clfA*/*B*), collagen binding adhesion (*can*), cell wall associated fibronectin binding protein (*ebh*), cell surface binding protein (*ebpS*), fibronectin-binding protein A/B (*fnb*A/*B*), major histocompatibility complex class II (extracellular adherence protein), extracellular adherence protein (*map*), and fibrinogen-/bone sialoprotein-binding protein C/D (*sdrC*/*D*)] were detected in all isolates yielding hybridization patterns corresponding to the respective clonal complexes.

## Discussion

*S. aureus* is an opportunistic pathogen in food producing animals and has been isolated from various animal species (Fluit, 2012). The incidence of MRSA in clinically healthy poultry might represent a relevant issue regarding to consumer protection. The results contribute to estimate this potential health hazards, it is imperative to know the pathogenic potential of MRSA isolates. For this reason, the study provided relevant information about MRSA from poultry based on molecular genotyping including detection of virulence factors and antimicrobial resistance determinants.

Mobile genetic elements (MGEs) encode putative virulence factors and molecules that confer resistance to antibiotics, including the gene that confers resistance to beta-lactam antibiotics in methicillin-resistant *S. aureus* (MRSA). Plasmids are auto-replicating DNA molecules. Staphylococci typically carry one or more plasmids per cell. Staphylococcal plasmids can carry a single resistance determinant, several resistance determinants or multi-resistance plasmids (Malachowa and DeLeo, 2010).

In the present study, MRSA isolated from clinically healthy poultry from different farms in Germany have been analyzed. Molecular genotyping of *S. aureus* isolated from turkey and broiler farms based on microarray hybridization assays resulted in assignment to two clonal complexes and three strains: CC5-MRSA-III, CC398-MRSA-V, and CC398-MRSA with unidentified/truncated SCC*mec* elements.

MRSA isolates from turkeys belonged to all of these three strains. While MRSA isolates from broilers belonged to clonal complex 398-MRSA-V and CC398-MRSA with unidentified/truncated SCC*mec* elements. This is in accordance with previous studies (Monecke et al., 2013) indicating that CC398-MRSA-V is a major *S. aureus* lineage in poultry and more abundant than other types. In general, this type is widespread and it has been detected in a wide range of host species from various countries (Monecke et al., 2007, 2011; Nemati et al., 2008; van Duijkeren et al., 2008; Lozano et al., 2009; Pan et al., 2009).

In this study, CC5-MRSA-III was isolated from turkey which has previously been isolated from Korean chicken meat samples (Kwon et al., 2006), German turkey meat products (Feßler et al., 2011) and diseased turkeys in Germany (Monecke et al., 2013). However, it was also observed among humans in KwaZulu-Natal/South Africa (Shittu et al., 2009).

All detected *spa* types (t002, t011, t034, t899, and t1430) were previously reported among MRSA isolates from turkey products and diseased turkeys (Bystroń et al., 2010; Feßler et al., 2011; Richter et al., 2012; Argudín et al., 2013; Monecke et al., 2013). *spa* type t034 is one of the most common type found in CC398 isolates in pigs (Hasman et al., 2010) but also of other origin, while t899 has been found in pig isolates (van Duijkeren et al., 2008), diseased turkeys (Argudín et al., 2013), and recently in healthy chickens in Belgium (Nemeghaire et al., 2013).

A weakness of current *spa*-typing primers is that rearrangements in the IgG-binding region of the gene cause 1–2% of strains to be designated as “non-typeable” (Votintseva et al., 2014).

For two CC398-MRSA, an amplification of *spa* repeat sequences was not possible, hence they were regarded as un-typable *spa*. Such isolates have been found before in diseased turkeys in Germany (Monecke et al., 2013).

Non-typeability of *S. aureus* strains can be attributed to deletions in the *spa*-gene, explaining the lack of amplification in some of MRSA samples. However, the persistence of mixed sequence traces that could not be resolved by typing individual colonies indicated the presence of other types of *spa*-gene rearrangements (Votintseva et al., 2014).

The *spa* t1403 associated with 398-MRSA-V has been previously reported for isolates from humans (Köck et al., 2013) and broiler flocks (Friese et al., 2013). CC5-MRSA-III with *spa* t002 was detected before in diseased turkeys (Monecke et al., 2013) and turkey meat (Feßler et al., 2011), but the *spa* type t002 is not indicative for this MRSA strain as it is abundant among CC5 isolates of any origin and SCC status.

Recently, a novel hybrid LA-MRSA CC9/CC398 genotype has been observed among persons living in urban areas of Denmark. This genotype had never been detected in Danish livestock. In contrast to Denmark, CC9/CC398 has been isolated from pigs, cattle, poultry, and retail foods in other European countries, including France, Germany, Italy, the Netherlands, and Spain. A subpopulation of CC9/CC398 has become adapted to humans and that poultry meat may serve as a vehicle for the transmission of such isolates (Larsen et al., 2016).

The different use of antimicrobial substances can influence the antimicrobial resistance patterns observed in bacterial populations. All isolated MRSA from both broilers and turkeys were tetracycline resistant and carried the penicillinase operon *bla*Z/I/R and the tetracycline resistance genes *tet*K/M which was in accordance with a previous study conducted in diseased turkeys (Argudín et al., 2013).

In this study, the MRSA isolates from broilers showed higher resistance rates to erythromycin in comparison to broiler isolates. High erythromycin resistance rate among MRSA isolated from broilers was demonstrated before in Germany (Feßler et al., 2011; Richter et al., 2012; Monecke et al., 2013), in Belgium (Nemeghaire et al., 2013) and the Netherlands (Wendlandt et al., 2013). In contrast, a previous report (Jaglic et al., 2012) demonstrated that erythromycin-resistant *S. aureus* colonized predominantly turkeys.

In this study, 55.6% of *S. aureus* isolated from turkey farms were resistant to erythromycin, while previous study conducted on 30 MSSA and one MRSA isolates from diseases turkeys revealed very low percentages of erythromycin resistance up to 9.7% (Argudín et al., 2013).

Eleven MRSA isolates (61.1%) from turkeys were resistant to streptogramin which in agreement with previous studies (Kadlec et al., 2009, 2010; Feßler et al., 2011).

The MRSA in this study showed the typical multi-drug resistance pattern as discussed in other reports concerning poultry isolates (Feßler et al., 2011; Richter et al., 2012; Monecke et al., 2013) and also isolates from swine and cattle (Kadlec et al., 2009; Feßler et al., 2010).

The carriage of virulence factors (including leukocidins, proteases, staphylococcal super-antigen-like proteins, and hemolysin genes) and adhesion factors were similar between the broiler and turkey isolates and resembled those of other German isolates originating from diseased turkeys (Monecke et al., 2013). According to previous studies, the presence of enterotoxin genes seems to be rare among CC398-MRSA isolates (Kadlec et al., 2009; Feßler et al., 2011). Enterotoxin genes of the *egc* locus are usually more common among nasal than invasive isolates of human origin, and it has been proposed that the *egc* encoded toxins could enhance the carriage of *S. aureus* in humans (van Belkum et al., 2006), but nothing has been stated on its role in animals. An incomplete *egc* cluster, with only the *seg* gene, has also been described in one human nasal isolate in Japan (Omoe et al., 2005) and one ST398 (Laurent et al., 2009). None of the MRSA isolates from turkeys or broilers was positive in the microarray investigation for enterotoxin B genes (*seb*), K (*sek*), and Q (*seq*) which have previously been detected in ST398-MRSA isolates from pigs (Kadlec et al., 2009) but not detected in MRSA isolated from food and food products (Feßler et al., 2011).

All isolates were positive for the presence of adhesion factors and genes encoding microbial surface components recognizing adhesive matrix molecules, especially *bbp* and *sdrD*. These genes appeared common among German MRSA isolates from poultry (Monecke et al., 2013) and in swine (Kadlec et al., 2009). In contrast, the absence of both genes (*bbp* and *sdrD*) was common in diseased turkeys (Argudín et al., 2013), methicillin-susceptible *S. aureus* (MSSA) isolates from turkeys in Germany (Monecke et al., 2013), and in Belgian MRSA isolates from poultry, pigs, horses, chicken and bovines (Nemati et al., 2009; Jamrozy et al., 2012).

In conclusion, this study was performed to gain insight into genotypic characteristics of MRSA isolated from healthy turkeys and broilers, their virulence factors, and their antimicrobial resistance patterns. Presented results concerning multidrug resistance are alarming and need further investigation.

## Author contributions

HE, MA, HH, RE, and HMH participated in the design of the study, performed the experiments, analyzed the data and drafted the manuscript. SM and JS participated in the experiments. HN and HMH participated in the design of the study and edited the manuscript. HE, SM, RE and HH helped to interpret the results and edited the manuscript. HE, MA, HH, SM, JS, JH, RE, HN and HMH coordinated the study, participated in the design of the study, helped to interpret the results and edited the manuscript. All authors read and approved the final manuscript.

### Conflict of interest statement

RE and SM are employees of Alere Technologies GmbH. The other authors declare that the research was conducted in the absence of any commercial or financial relationships that could be construed as a potential conflict of interest.
