# Supplementary material for: Characterization of Methicillin-Resistant Staphylococcus aureus Isolated from Healthy Turkeys and Broilers Using DNA Microarrays
Source: Front Microbiol. 2016 Dec 19;7:2019. doi: 10.3389/fmicb.2016.02019 (PMC5165244; doi:10.3389/fmicb.2016.02019)
Supplement: Supplementary file 1 [file DataSheet1.DOCX]

**Supplementary File:** Distribution of detected resistance and/or virulence genes encoded on chromosome or plasmid

| **symbol** | **variability** | **product** |
| --- | --- | --- |
| **aacAaphD** | plasmidic | bifunctional enzyme Aac/Aph |
| **aadD** | plasmidic (can be integrated into SCC) | aminoglycoside adenyltransferase |
| **agrB** | core variable | accessory gene regulator B |
| **agrC** | core variable | accessory gene regulator C |
| **agrD** | core variable | accessory gene regulator D |
| **aphA** | mobile (plasmid/transposon) | 3'5'-aminoglycoside phosphotransferase |
| **arcA/B/C/D-SCC** | SCC element | ornithine carbamoyltransferase locus 3 |
| **aur** | core variable | aureolysin, supergroup 1 |
| **bap** | genomic island | surface protein involved in biofilm formation |
| **bbp** | core hypervariable | bone sialoprotein-binding protein |
| **blaI** | plasmidic | beta-lactamase repressor inhibitor |
| **blaR** | plasmidic | beta-lactamase regulatory protein |
| **blaZ** | plasmidic | beta-lactamase |
| **blaZ-SCCmec XI** | SCC element | putative beta-lactamase |
| **capH1** | genomic island | capsular polysaccharide biosynthesis protein Cap1H |
| **capH5** | genomic island | capsular polysaccharide biosynthesis protein Cap5H |
| **capH8** | genomic island | capsular polysaccharide synthesis enzyme Cap8H |
| **capI8** | genomic island | capsular polysaccharide synthesis enzyme Cap8I |
| **capJ1** | genomic island | capsular polysaccharide synthesis enzyme Cap1J |
| **capJ5** | genomic island | capsular polysaccharide biosynthesis protein Cap5J |
| **capJ8** | genomic island | capsular polysaccharide synthesis enzyme Cap8J |
| **capK1** | genomic island | capsular polysaccharide synthesis enzyme Cap1K |
| **capK5** | genomic island | capsular polysaccharide biosynthesis protein Cap5K |
| **capK8** | genomic island | capsular polysaccharide synthesis enzyme Cap8K |
| **cat** | plasmidic | chloramphenicol acetyltransferase |
| **cat-pC221** | plasmidic | chloramphenicol acetyltransferase |
| **ccrA-1** | SCC element | cassette chromosome recombinase A, type 1 |
| **ccrA-2** | SCC element | cassette chromosome recombinase A, type 2 |
| **ccrA-3** | SCC element | cassette chromosome recombinase A, type 3 |
| **ccrA-4** | SCC element | cassette chromosome recombinase A, type 4 |
| **ccrA-4** | SCC element | cassette chromosome recombinase A, type 4 |
| **ccrA-5** | SCC element | cassette chromosome recombinase A, type 5 |
| **ccrAA** | SCC element | cassette chromosome recombinase AA |
| **ccrB-1** | SCC element | cassette chromosome recombinase B, type 1 |
| **ccrB-2** | SCC element | cassette chromosome recombinase B, type 2 |
| **ccrB-3** | SCC element | cassette chromosome recombinase B, type 3 |
| **ccrB-4** | SCC element | cassette chromosome recombinase B, type 4 |
| **ccrC** | SCC element | cassette chromosome recombinase C |
| **cfr** | mobile | 23S rRNA methyltransferase |
| **chp** | mobile | chemotaxis-inhibiting protein (CHIPS) |
| **clfA** | core hypervariable | clumping factor A |
| **clfB** | core hypervariable | clumping factor B |
| **cna** | genomic island | collagen-binding adhesin |
| **coa** | core hypervariable | staphylococcal coagulase |
| **dfrA** | plasmidic | dihydrofolate reductase |
| **ear2** | genomic island | putative protein |
| **ebh** | genomic island | cell wall associated fibronectin-binding protein |
| **ebpS** | core variable | cell surface elastin binding protein |
| **edinA** | plasmidic | epidermal cell differentiation inhibitor A |
| **edinB** | mobile | epidermal cell differentiation inhibitor B |
| **edinC** | plasmidic | epidermal cell differentiation inhibitor C |
| **efb** | core variable | extracellular fibrinogen-binding protein |
| **eno** | core genome | enolase |
| **entA** | mobile | enterotoxin A, strain FRI100 |
| **entA-320E** | mobile | enterotoxin A, strain 320E |
| **entA-N315** | mobile | enterotoxin A of strain N315 |
| **entB** | mobile | enterotoxin B |
| **entC** | mobile | enterotoxin C |
| **entC** | mobile | enterotoxin C |
| **entD** | plasmidic | enterotoxin D |
| **entG** | genomic island | enterotoxin G |
| **entH** | genomic island | enterotoxin H |
| **entI** | genomic island | enterotoxin I |
| **entJ** | plasmidic | enterotoxin J |
| **entK** | mobile | enterotoxin K |
| **entL** | mobile | enterotoxin L |
| **entM** | genomic island | enterotoxin M |
| **entN** | genomic island | enterotoxin N |
| **entO** | genomic island | enterotoxin O |
| **entQ** | mobile | enterotoxin Q |
| **entR** | plasmidic | enterotoxin R |
| **entU** | genomic island | enterotoxin U, enterotoxin Y |
| **entV** | genomic island | enterotoxin V chimera |
| **ermA** | mobile (plasmidic/can be integrated into SCC) | rRNA adenine N-6-methyltransferase |
| **ermB** | plasmidic | rRNA methyltransferase |
| **ermC** | plasmidic | rRNA adenine N-6-methyltransferase |
| **etA** | mobile | exfoliative toxin serotype A |
| **etB** | plasmidic | exfoliative toxin serotype B |
| **etD** | mobile | exfoliative toxin D |
| **fexA** | plasmidic | chloramphenicol/florfenicol exporter |
| **fnbA** | core hypervariable | fibronectin-binding protein A |
| **fnbB** | genomic island | fibronectin-binding protein B |
| **fosB** | genomic island | fosfomycin resistance protein |
| **fosB-pla** | plasmidic | fosfomycin resistance protein |
| **fusB** | plasmidic | fusidic acid resistance protein B |
| **fusC** | SCC element | fusidic acid resistance protein C |
| **gapA** | core genome | glyceraldehyde 3-phosphate dehydrogenase, locus A |
| **hla** | core variable | hemolysin alpha |
| **hlb** | core genome | hemolysin beta |
| **hld** | core genome | hemolysin delta |
| **hlgA** | core genome | hemolysin gamma component A |
| **hlIII** | core variable | channel protein, hemolysin III family protein |
| **hsdS (all alleles)** | SCC element | type I restriction-modification system site-specificity determinate |
| **hysA (all alleles)** | core genome | hyaluronate lyase |
| **icaA** | core variable | intercellular adhesion protein A |
| **icaC** | core variable | intercellular adhesion protein C |
| **icaD** | core variable | intercellular adhesion protein D |
| **isaB** | core variable | immunodominant antigen B |
| **isdA** | core variable | extracellular transferrin-binding protein |
| **katA** | core variable | catalase A |
| **kdpA-SCC** | SCC element | potassium-transporting ATPase A, chain 2 |
| **kdpB-SCC** | SCC element | potassium-transporting ATPase B, chain 1 |
| **kdpC-SCC** | SCC element | potassium-transporting ATPase C, chain 2 |
| **kdpD-SCC** | SCC element | two component sensor/regulator of kdp operon, sensor histidine kinase |
| **kdpE-SCC** | SCC element | two component sensor/regulator of kdp operon, transcriptional regulator |
| **lmrP** | core variable /plasmidic | integral membrane transporter, group 1 |
| **lnuA** | plasmidic | lincosamide nucleotidyltransferase |
| **lukD** | genomic island | leukocidine D |
| **lukE** | genomic island | leukocidine E |
| **lukF** | core variable | hemolysin gamma component B |
| **lukF-PV** | mobile | Pantone-Valentine leukocidine subunit F |
| **lukF-PV83** | mobile | bovine Pantone-Valentine leukocidine F |
| **lukM-PV83** | mobile | bovine Pantone-Valentine leukocidine S |
| **lukS** | core variable | hemolysin gamma component C |
| **lukS-PV** | mobile | Pantone-Valentine leukocidine subunit S |
| **lukX** | core variable | leukocidin/hemolysin toxin family protein |
| **lukY** | core variable | leukocidin/hemolysin toxin family protein |
| **map** | core variable | major histocompatibility complex class II analog protein |
| **mecA** | SCC element | penicillin binding protein 2a |
| **mecC** | SCC element | penicillin binding protein 2a |
| **mecI** | SCC element | meticillin-resistance regulatory protein |
| **mecR1** | SCC element | meticillin resistance operon repressor 1 |
| **mecR2** | SCC element | meticillin resistance operon repressor 2 |
| **mecR2-Sxyl** | core genome | meticillin resistance operon repressor 2 |
| **mefA** | mobile | macrolide efflux protein A |
| **merA** | plasmidic (can be integrated into SCC) | mercury reductase |
| **merB** | plasmidic (can be integrated into SCC) | alkylmercury lyase |
| **mpbBM** | plasmidic | macrolide 2'-phosphotransferase II |
| **mprF** | core variable | lysylphosphatidylglycerol synthetase |
| **msrA** | plasmidic | energy-dependent efflux of erythromycin |
| **mupA** | plasmidic | plasmidic isoleucyl-tRNA synthase |
| **nuc1** | core variable | thermostable extracellular nuclease locus 1 |
| **ORF CM14** | genomic island | enterotoxin-like protein |
| **plsSCC-COL** | SCC element | plasmin-sensitive surface protein |
| **Q2YUB3** | mobile | multidrug resistance transporter |
| **Q7A4X2** | genomic island | putative protein |
| **Q9XB68-dcs** | SCC element | putative protein |
| **qacAB** | plasmidic | quaternary ammonium compound resistance protein A and B |
| **rnaIII** | core genome | regulatory RNA III |
| **rrlD1** | core genome | 23S rRNA domain 1 |
| **saeS** | core genome | histidine protein kinase, sae locus |
| **sak** | mobile | staphylokinase |
| **sarA** | core genome | staphylococcal accessory regulator A |
| **sasG** | genomic island | Staphylococcus aureus surface protein G |
| **sat** | mobile (plasmid/transposon) | streptothricine-acetyltransferase |
| **sbi** | core variable | staphylococcal IgG-binding protein |
| **scn** | mobile | staphylococcal complement inhibitor |
| **sdrC** | core hypervariable | serine aspartate repeat protein C |
| **sdrD** | genomic island | serine aspartate repeat protein D |
| **sdrM** | core variable | multidrug efflux protein |
| **setB1** | core variable | staphylococcal enterotoxin-like toxin B locus 1 |
| **setB2** | core variable | staphylococcal enterotoxin-like toxin B locus 2 |
| **setB3** | core variable | staphylococcal enterotoxin-like toxin B locus 3 |
| **setC** | genomic island | staphylococcal enterotoxin-like toxin X |
| **smr** | plasmidic | quaternary ammonium compound resistance protein |
| **spa** | core hypervariable | immunoglobin G binding protein A |
| **splA** | genomic island | serine protease A |
| **splB** | genomic island | serine protease B |
| **splE** | genomic island | serine protease E |
| **ssl01** | genomic island | staphylococcal superantigene like protein locus 1 |
| **ssl02** | genomic island | staphylococcal superantigene like protein locus 2 |
| **ssl03** | genomic island | staphylococcal superantigene like protein locus 3 |
| **ssl04** | genomic island | staphylococcal superantigene like protein locus 4 |
| **ssl05** | genomic island | staphylococcal superantigene like protein locus 5 |
| **ssl06** | genomic island | staphylococcal superantigene like protein locus 6 |
| **ssl07** | genomic island | staphylococcal superantigene like protein locus 7 |
| **ssl08** | genomic island | staphylococcal superantigene like protein locus 8 |
| **ssl09** | genomic island | staphylococcal superantigene like protein locus 9 |
| **ssl10** | genomic island | staphylococcal superantigene like protein locus 10 |
| **ssl11** | genomic island | staphylococcal superantigene like protein locus 11 |
| **sspA** | core hypervariable | glutamyl endopeptidase |
| **sspB** | core variable | staphopain B |
| **sspP** | genomic island | staphopain A |
| **tetK** | plasmidic | tetracycline efflux protein variant K |
| **tetM** | mobile (plasmidic/can be integrated into SCC) | ribosomal protection tetracycline resistance protein, chimera |
| **tst** | mobile | toxic shock syndrome toxin-1 |
| **ugpQ** | SCC element | glycerophosphoryl diester phosphodiesterase |
| **vanA** | mobile | vancomycin resistance protein A |
| **vanB** | mobile | vancomycin resistance protein B |
| **vanZ** | plasmidic | vancomycin resistance protein Z |
| **vatA** | plasmidic | virginiamycin A acetyltransferase |
| **vatB** | plasmidic | acetyltransferase inactivating streptogramin A |
| **vga** | plasmidic | streptogramin A ABC transporter |
| **vga-A** | mobile | streptogramin A ABC transporter |
| **vga-B** | mobile | streptogramin A ABC transporter |
| **vga-C** | plasmidic | streptogramin A ABC transporter |
| **vga-D** | mobile | streptogramin A ABC transporter |
| **vga-E** | mobile | streptogramin A ABC transporter |
| **vgb** | plasmidic | virginiamycin B hydrolase |
| **vraS** | core genome | two component sensor/regulator, sensor histidine kinase |
| **vwb** | core hypervariable | "van Willebrand factor" binding protein |
| **vwb3** | mobile | "van Willebrand factor" binding protein |
